# Impact of Pulmonary Venous Inflow on Cardiac Flow Simulations: Comparison with *In Vivo* 4D Flow MRI

**DOI:** 10.1007/s10439-018-02153-5

**Published:** 2018-10-24

**Authors:** Jonas Lantz, Vikas Gupta, Lilian Henriksson, Matts Karlsson, Anders Persson, Carl-Johan Carlhäll, Tino Ebbers

**Affiliations:** 1 Division of Cardiovascular Medicine, Department of Medical and Health Sciences, Linköping University, Linköping, Sweden; 2 Center for Medical Image Science and Visualization (CMIV), Linköping University, Linköping, Sweden; 3 Division of Radiology, Department of Medical and Health Sciences, Linköping University, Linköping, Sweden; 4 Division of Applied Thermodynamics and Fluid Mechanics, Department of Management and Engineering, Linköping University, Linköping, Sweden; 5 Department of Clinical Physiology, Department of Medical and Health Sciences, Linköping University, Linköping, Sweden

**Keywords:** Sensitivity analysis, Design-of-experiments, Computational fluid dynamics, *In vivo* measurements

## Abstract

Blood flow simulations are making their way into the clinic, and much attention is given to estimation of fractional flow reserve in coronary arteries. Intracardiac blood flow simulations also show promising results, and here the flow field is expected to depend on the pulmonary venous (PV) flow rates. In the absence of *in vivo* measurements, the distribution of the flow from the individual PVs is often unknown and typically assumed. Here, we performed intracardiac blood flow simulations based on time-resolved computed tomography on three patients, and investigated the effect of the distribution of PV flow rate on the flow field in the left atrium and ventricle. A design-of-experiment approach was used, where PV flow rates were varied in a systematic manner. In total 20 different simulations were performed per patient, and compared to *in vivo* 4D flow MRI measurements. Results were quantified by kinetic energy, mitral valve velocity profiles and root-mean-square errors of velocity. While large differences in atrial flow were found for varying PV inflow distributions, the effect on ventricular flow was negligible, due to a regularizing effect by mitral valve. Equal flow rate through all PVs most closely resembled *in vivo* measurements and is recommended in the absence of *a priori* knowledge.

**Electronic supplementary material:**

The online version of this article (10.1007/s10439-018-02153-5) contains supplementary material, which is available to authorized users.

## Introduction

Complementary to *in vivo* flow measurements, computational flow models based on high-resolution computed tomography (CT) can provide detailed information on blood flow characteristics, such as flow instabilities, pressure distribution, or blood residence time.^4^,^5^,^23^,^28^ Current CT technology is able to acquire time-resolved anatomy on a sub-millimeter level, making CT-based computational models ideal for flow studies of cardiac geometry. The heart motion can be extracted from clinical image data and prescribed in the model, effectively creating a one-way transfer of momentum from the moving endocardium to the blood volume.

Currently, flow simulation studies are mainly focused on the left ventricle (LV), while the LA is often ignored or significantly simplified. Blood flow in the left atrium (LA) is complex.^6^,^13^,^25^,^28^ Multiple vortices form as blood from the four pulmonary veins (PVs) collides in the LA, before being pulled through the mitral valve into the left ventricle. Abnormal hemodynamics could potentially explain the initiation and progression of thrombus formation in both the LA and the left atrial appendage (LAA).^1^,^16^,^17^,^21^ Clinically, LA hemodynamics is normally assessed by Doppler ultrasound^15^,^26^ which is limited to flow measurements in one direction, and assessment focusses therefore mainly on inflow from the pulmonary veins and left atrial appendage. Three-dimensional time-resolved flow magnetic resonance imaging, popularly called 4D flow MRI, enable volumetric assessment of the intra-cardiac flow patterns.^10^ However, spatial and temporal resolution are usually in the order of 2.5 × 2.5 × 2.5 mm and 40 ms, respectively, which can be insufficient for studies of the LA as both the PVs and LAA can be small and moving fast.

In simulations, the atrium is frequently replaced with a mock geometry (pipe or simplified chamber) or simply by a time-varying flow boundary condition.^7^,^8^,^20^,^24^,^27^,^28^ Small changes in boundary conditions are known to affect the computed flow patterns,^24^ but the effect of these simplified geometries on ventricular blood flow patterns is debated. Recently, a computational study^28^ investigated the effect on ventricular flow when replacing a physiological LA model with a pipe model. Velocity differences in the LV of about 10% between their physiological and simplified model were reported, and it was concluded that strong vortex dissipation in the LA and a regularizing effect by the mitral valve contributed to the small difference in LV velocities. This is in contrary to an earlier study^25^ who performed similar simulations and found that vorticity produced in the LA by the PVs were transported into the LV through the mitral valve and significantly affected LV diastolic flow patterns. Neither studies compared their results with *in vivo* measurements.

When the LA is included in a computational model, the flow through each PV must be accounted for. Patient-specific *in vivo* flow measurements could potentially be used, but are rarely available. The total inflow rate through all four pulmonary veins can be calculated *a priori* from medical image data as the time-rate-of-change of the cardiac blood pool volume, but individual PV flow rates cannot be determined directly, and some sort of assumption must be made. One approach is to prescribe 25% of the total pulmonary flow rate on each PV.^4^,^5^ In absence of *in vivo* measurements this is a straight-forward but rather simplistic approach, as virtually any other flow combination is possible.

In a previous study, we computed intracardiac blood flow based on CT on twelve patients with suspected heart disease, and compared the computed results with *in vivo* 4D Flow MRI measurements on the same patients.^22^ There, 25% of the total flow was assumed to enter the LA through each PV, and while very good agreement with *in vivo* measurements was found, a sensitivity analysis on inlet flow rates was not performed.

In this study we investigated the sensitivity of intracardiac blood flow dynamics for different pulmonary venous inflow rates. We performed CT-based computational flow simulations on three patients with suspected heart disease, and compared results to *in vivo* 4D Flow MRI measurements of the same patients. A Design of Experiment (DoE) analysis was performed where the distribution of the blood flow between pulmonary veins was changed in a systematic manner, resulting in 60 different simulations. Flow features in the LA and LV were studied separately, in order to investigate any regularizing effect from the mitral valve on ventricular blood flow.

## Materials and Methods

### Patient Population

The patients included in this study are taken from an earlier study where we computed intracardiac blood flow based on CT and compared results with *in vivo* flow measurements.^22^ In addition to 4D Flow MRI measurements of the LA and LV, the PVs were also available in the 4D flow MRI data for three of those patients, and they were included in this study. The three patients had a clinical referral for coronary CT angiography due to suspected coronary artery disease. All patients had similar heart rates during both image acquisitions, see Table 1. The CT images were used as input for the simulation model, while the MRI data was solely used for the comparison with simulation results. Written informed consent was obtained from all patients and the study was approved by the local ethics review board at Linköping University Hospital.

**Table 1:** Data on the patents included in the study.

Patient	Age	Sex	Height	Weight	BP	HR CT	HR MRI	*β*-blockers	LVEDV	LVESV	LVEF	CT-MRI
#1	62	F	163	70	139/83	60	58	No	94	42	56	<2 h
#2	57	M	174	80	158/76	63	63	Yes	100	38	62	1 week
#3	66	F	163	83	130/75	66	65	No	113	51	54	<2 h

Age in (years), sex (male/female), height in (cm), weight in (kg), blood pressure in (mmHg), heart rate during CT (bpm), heart rate during MRI (bpm), if beta-blockers were administered, *LVEDV* left ventricular end diastolic volume in (mL) measured by CT, *LVESV* left ventricular end systolic volume in (mL) measured by CT, *LVEF* left ventricular ejection fraction in (%), *CT-MRI* time between CT and MRI acquisitions (hours or weeks)

### Image Acquisition and Registration

CT image acquisition was performed using a third-generation dual source CT (Siemens SOMATOM Force, Siemens Medical Solutions, Germany). Acquisition parameters were as follows: Detector collimation: 192 × 0.6 mm, Gantry rotation time: 0.25 s, Pitch: 0.15–0.34, Quality reference: 276 mA s, Reference kV: 100 kV. Data was acquired during a single inspiration-breath hold. Retrospective image acquisition with ECG-triggered dose modulation was used, and 20 phases between two R–R intervals were reconstructed. The reconstructed slice thickness was 0.5 mm with a 0.25 mm increment and in-plane resolution was 0.35 × 0.35 ± 0.06 mm, depending on patient. Cardiac geometry was manually segmented in a single time frame and used as input to an in-house image registration framework. The framework tracked the wall motion over the cardiac cycle, and was validated against manually segmented geometries.^14^ For details on the image registration framework, see earlier work.^22^ The extracted wall motion was then prescribed in a flow solver—see “Computational Fluid Dynamics”.

The MRI acquisition was performed using a clinical 3T scanner (Philips Ingenia, Philips Healthcare, The Netherlands). 4D Flow data was acquired at end-expiration during free-breathing using navigator-based respiratory gating of a gradient-echo pulse-sequence with interleaved three-directional flow-encoding and retrospective vector cardiogram controlled cardiac gating. Scan parameters were as follows: VENC: 120 cm/s, Flip Angle: 5°, Echo Time: 2.9 ms, Repetition Time: 5.0 ms, TFE factor: 2. The acquired spatial resolution was 2.9 × 2.9 × 2.9 mm and effective acquired temporal resolution 40 ms. Morphological images in 2-, 3-, and 4-chamber views together with 4D Flow data were acquired.

### Computational Fluid Dynamics

**Figure 1:**
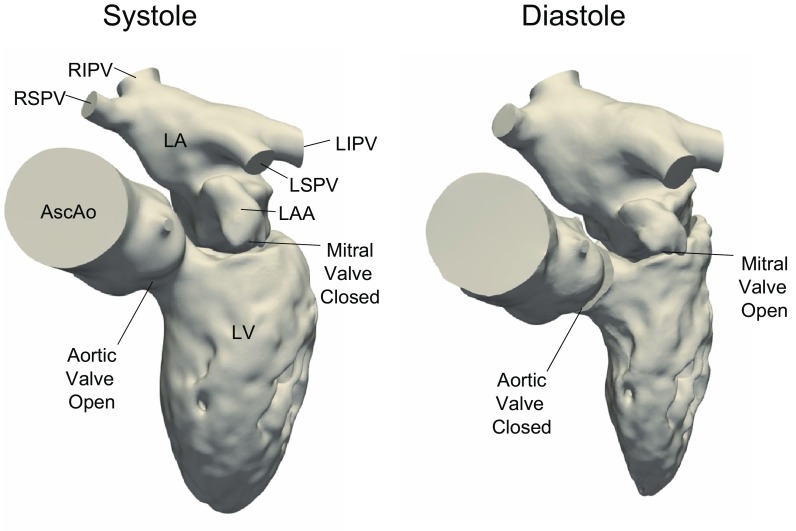
Geometry of one of the patients at early systole and early diastole. During systole when the mitral valve is closed, the LA and LV are topologically separated. During diastole the mitral valve is open and the LA and LV are topologically connected. *LA* Left atrium, *LAA* left atrial appendage, *RSPV/RIPV* right superior and inferior pulmonary veins, *LSPV/LIPV* left superior and inferior pulmonary veins, *LV* left ventricle, *AscAo* ascending aorta.

The methodology has been presented in detail in previous work,^22^,^23^ but is briefly presented here for completeness. The geometries used in the simulations included the pulmonary veins, left atrium, mitral valve, left ventricle with papillary muscles and trabeculae, aortic valve and ascending aorta, see Fig. 1. Heart valves were considered to be either opened or closed, but moved with the valve plane. Using the extracted wall motion from the CT acquisition, deformed cardiac geometries were generated every 10 ms for the entire cardiac cycle. Based on those geometries the flow field was computed using CFX 17.0 (Ansys, USA). No turbulence modelling was applied, as initial simulations showed no significant flow instabilities, and no significant turbulent kinetic energy levels were measured by 4D Flow MRI. The temporal resolution was 500 *µ*s and spatial resolution was in the range of 9–14 million computational cells, with the smallest length scale on the order of 50 *µ*m. Numerical schemes were second-order accurate, and blood was simulated as an incompressible fluid with density 1060 kg/m^3^ and viscosity 3.5e-3 Pa s. Data were saved every 10 ms. Simulation time was approximately 6–10 h per cardiac cycle using 96 CPU cores (Intel Xeon E5-2660 Sandy Bridge processors at 2.2 GHz).

#### Boundary Conditions

The wall motion was extracted directly from the CT images, effectively creating a one-way transfer of momentum from the endocardium to the blood. Thus, the intracardiac flow did not affect the motion of the heart wall, and was determined entirely by the wall motion and inlet flow boundary conditions. As the flow through the aortic valve was determined solely by LV volume change, a pressure boundary condition with zero relative pressure was used at the ascending aorta. Similarly, the total pulmonary vein inflow rate, *Q*_PV_, into the LA through the four PVs was directly determined by the volumetric change of the geometry. During ventricular systole, when the mitral valve was closed, *Q*_PV_ was determined by the time derivative of the left atrial volume, and during systole when the mitral valve was open, *Q*_PV_ was defined as the sum of the time derivatives of the left atrial and left ventricular volumes:

1 $$Q_{\text{PV}} = \left\{ {\begin{array}{*{20}l} {\left. {\frac{{{\text{d}}V}}{{{\text{d}}t}}} \right|_{\text{LA}} } & {{\text{During}} \;{\text{systole}}} \\ {\left. {\frac{{{\text{d}}V}}{{{\text{d}}t}}} \right|_{\text{LA}} + \,\, \left. {\frac{{{\text{d}}V}}{{{\text{d}}t}}} \right|_{\text{LV}} } & {{\text{During}}\; {\text{diastole}}} \\ \end{array} } \right.,$$

where *V* represents left atrial and left ventricular volumes, respectively. Normally, there are four pulmonary veins acting as flow inlets to the atrium: the left and right superior and inferior pulmonary veins (LSPV, LIPV, RSPV, RIPV). As no flow information were available from the CT data, an assumption on local flow rate through each individual PV had to be made. The individual flow rate at each PV can be set as a fraction *f* of *Q*_PV_, and without *a priori* knowledge on flow rate distribution, an equal amount of flow was initially assumed to enter through each pulmonary vein, i.e. *f*_RSPV_= *f*_RIPV_= *f*_LIPV_= *f*_LSPV_= 25%.

### Design of Experiment Analysis

The sensitivity of prescribing an equal amount of flow through each PV was further explored by performing a design of experiment (DoE) analysis. The DoE concept is a strategy to maximize information output while using the least amount of experimental points. The flow inlet fractions *f*_RSPV_, *f*_RIPV_, *f*_LSPV_, and *f*_LIPV_ representing the fraction of total flow through each PV were set as design variables. The design space for the four input variables spans a 4-dimensional hypercube, and possible design parameters for each variable were generated using an optimal space-filling Sobol sequence.^9^ Hence, each point in the design space is at an optimal location, and subsequent addition of more design points will still be at an optimal location. The input variables were allowed to take on any value between 0 and 50%, as long as the sum of all four input variables were 100%. In total, 20 DoE parameter sets were computed, and are presented in Table 2. A few notes are necessary here: the first case (DoE #1) represents an equal flow fraction through each pulmonary vein (25% of the total flow rate in each PV), while DoE # 6 represent one extreme case where most of the flow was entering the LA through the superior pulmonary veins (41 and 48%) with almost no flow entering through the inferior pulmonary veins (5 and 6%). All 20 parameter sets were run for the three patients, resulting in 60 simulations with different inlet boundary conditions.

**Table 2:** Design of experiment analysis input variables. Each value represents the fraction in % of the instantaneous total flow rate through each pulmonary vein.

DoE #	*f*_RSPV_	*f*_RIPV_	*f*_LSPV_	*f*_LIPV_
1	25.0	25.0	25.0	25.0
2	12.5	37.5	12.5	37.5
3	37.5	12.5	37.5	12.5
4	23.4	4.8	23.4	48.4
5	16.0	30.1	23.0	30.9
6	41.0	5.1	48.0	5.9
7	48.8	9.8	24.6	16.8
8	16.8	16.8	25.4	41.0
9	41.8	41.8	0.4	16.0
10	23.0	48.0	19.2	9.8
11	3.7	32.3	23.6	40.4
12	28.7	7.2	48.7	15.4
13	35.0	26.0	4.9	34.1
14	47.5	13.5	17.3	21.7
15	8.7	19.6	29.0	42.7
16	33.7	44.6	4.0	17.7
17	21.2	32.1	41.5	5.2
18	46.2	7.1	16.5	30.2
19	27.1	6.7	26.3	39.9
20	14.1	26.7	30.5	28.7
Avg	27.6	22.4	24.1	26.0
Std	13.1	13.7	12.9	12.9

Average and standard deviation values for each vessel are presented at the bottom

*RSPV* right superior, *RIPV* right inferior, *LSPV* left superior and *LIPV* left inferior pulmonary vein

### Assessment of Results

The resulting flow rates through all PVs and mitral valve were compared to *in vivo* 4D Flow MRI measurements. For the MRI acquisition, streamlines were emitted in the LA and traced backwards to find the location of the PVs. Cross-sectional planes were then placed and the velocity integrated to obtain the flow rate. Intracardiac kinetic energy (KE) has in several studies shown to be correlated to the initiation and progression of different cardiac diseases.^2^,^3^,^11^,^12^,^18^ KE for LA and LV was computed as:

2 $${\rm KE} = \frac{\rho V}{2}v^{2} ,$$

where *ρ* is density of blood, *V* is the computational cell or MRI voxel volume, and *v* the velocity magnitude. The flow profile at the mitral annulus was investigated as this flow profile is commonly used as boundary condition in cardiac flow models and minor variations could potentially affect ventricular flow. Mitral flow profiles were extracted from the MRI data using an in-house tool and compared to the simulation results at early and late diastolic filling Velocity magnitude and short in-plane streamlines were used to indicate in-plane flow direction. To assess the variation in mitral valve profiles in the DoE analysis, mean and standard deviation of the velocity profiles were computed, and also compared to *in vivo* 4D Flow MRI measurements. Furthermore, based on DoE # 1 as baseline (*f *= 25%), root-mean-square-errors of the velocity magnitude in the entire model were calculated as:

3 $${\text{RMSE}} = \sqrt {\frac{1}{n}\mathop \sum \limits_{i = 1}^{n} (v_{25\% } - v_{i} )^{2} }$$

where *n* = 20 DoE cases and *v*_25%_ represents velocity magnitude for the baseline case. Results were visualized at contour planes covering the LA and LV at peak systole, early, and late diastolic filling. Volume-averages of RMSE for the LA and LV were computed separately for the whole cardiac cycle.

## Results

### Pulmonary Vein Flow Rates

**Figure 2:**
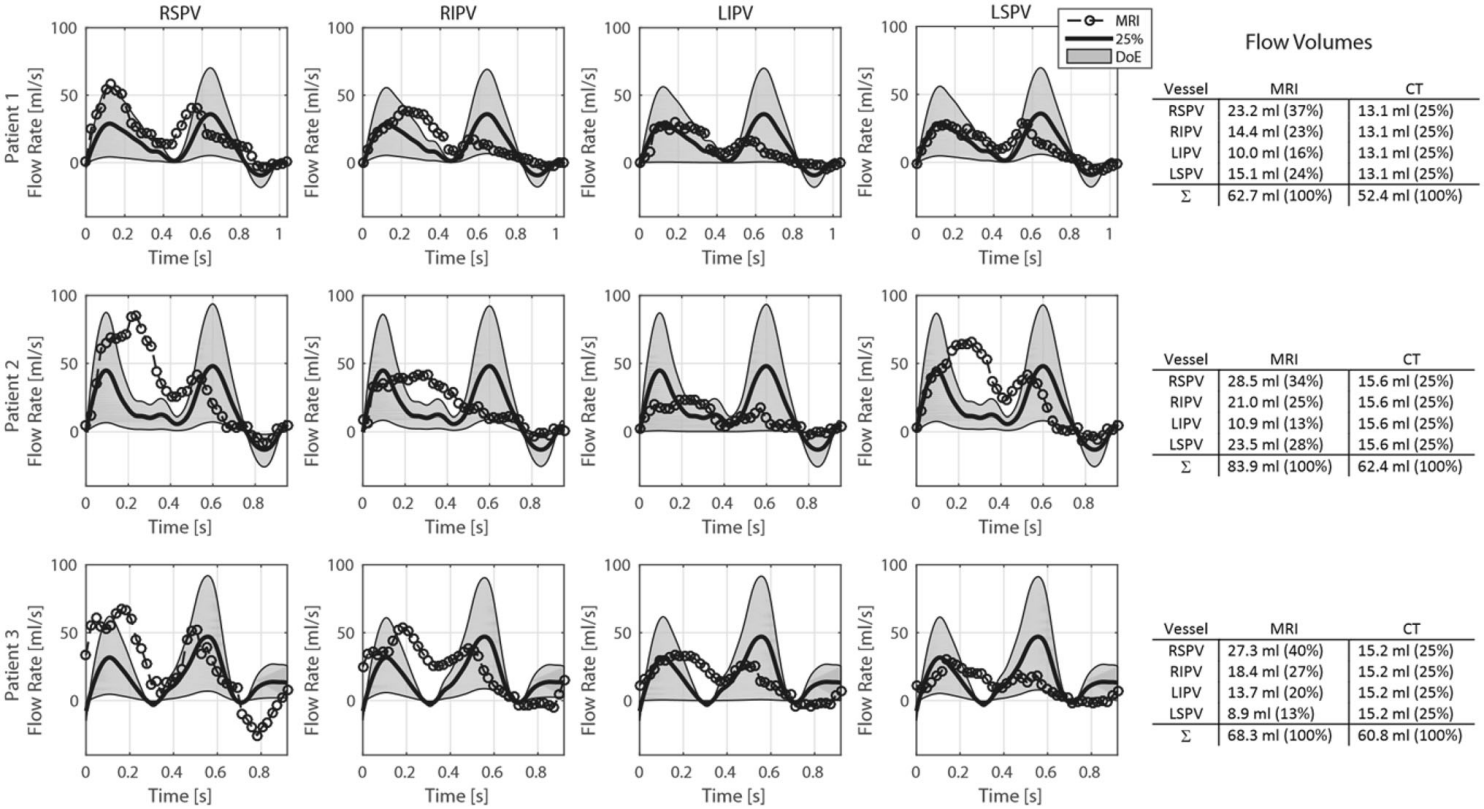
Measured and derived flow rates through each pulmonary vein for the three patients in the study. Dashed lines with circles represent flow rates from 4D Flow MRI measurements. Solid line represents 25% of the total incoming flow rate, derived from the total volume change of the CT-based geometry. Filled gray area represents possible solutions from the design-of-experiment analysis, where the inlet fractions *f* where changed from 25% to values between 0 and 50%, see Table 2. The resulting flow volumes through each pulmonary vein for both 4D Flow MRI measurements and CT-based simulations are reported in the table to the right. *RSPV* right superior, *RIPV* right inferior, *LIPV* left inferior, *LSPV* left superior pulmonary vein.

Flow rates at the right superior (RSPV), right inferior (RIPV), left inferior (LIPV) and left superior (LSPV) pulmonary veins were extracted from the 4D Flow MRI data and are presented together with the CT-based simulations with 25% flow distribution (denoted 25%) and all possible combinations of the DoE analysis (filled gray area, denoted DoE) in Fig. 2. Generally, a biphasic filling pattern could be observed, with the first inflow phase representing atrial filling during ventricular systole, and a second filling phase during early ventricular filling phase. Flow reversal at the PVs were observed during the late part of diastole for all patients in the MRI data, and for two patients in the CT-based simulations. The total flow volumes were consistently lower in the CT-based simulation compared to 4D Flow MRI measurements. MRI results showed consistently higher flow volumes for the right side PVs than the left side, and two patients had a larger flow volume in the superior PVs than the inferior PVs.

### Intra-Atrial Kinetic Energy

**Figure 3:**
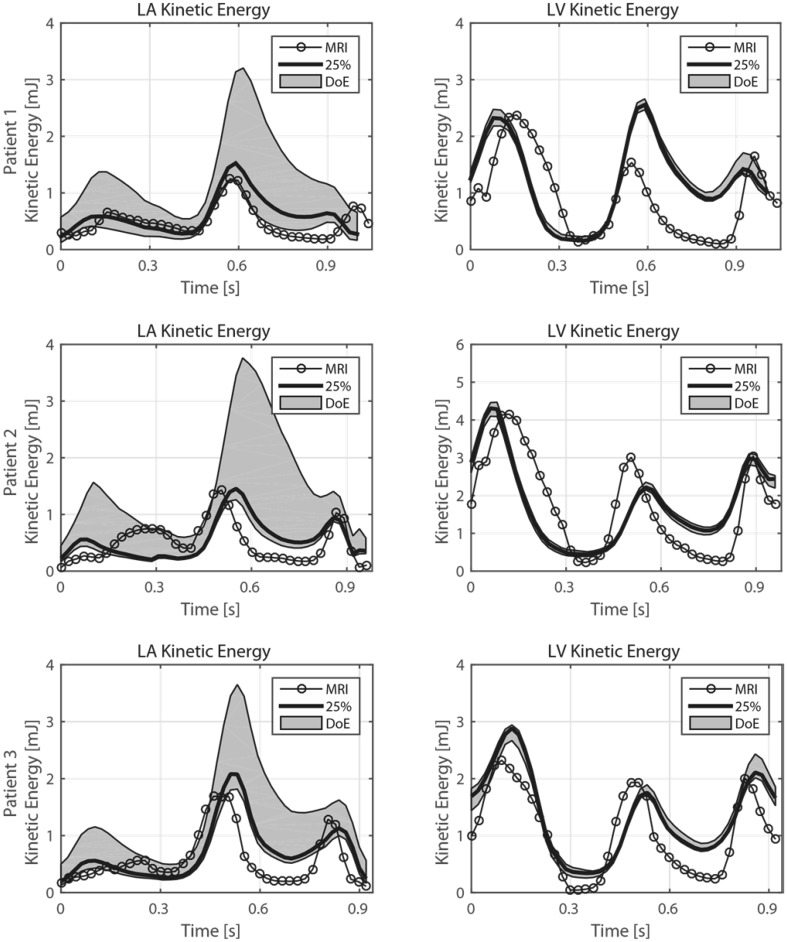
Integrated kinetic energy levels in the LA and LV for the three patients. The gray shaded area represents results from the DoE analysis (20 simulations), showing large variations in LA kinetic energy (KE) levels, as a result of the different inflow rates through the pulmonary veins. In contrast, LV KE-levels are more coherent and not as affected by the pulmonary vein flow rate.

The KE inside the LA and LV was computed for the *in vivo* 4D Flow MRI measurement and CT-based simulations, and are presented in Fig. 3. For the LA three peaks emerge, representing atrial filling during ventricular systole, and early and late ventricular diastolic filling. The curves are similar to the PV inflow curved presented in Fig. 2, with the highest KE values during the early diastolic filling phase. Comparing 4D Flow MRI measurements to the CT-based simulation with 25% inflow through each PV, similar KE values appear throughout the cardiac cycle. In contrast, results from the 20 DoE simulations (gray filled area) show large variations, which was expected. For some simulations in the DoE matrix most of the flow entered the LA through two PVs, resulting in high-velocity jets, effectively increasing the LA KE. It is evident from Fig. 3 that an equal amount of flow through each PV results in low KE in the LA, while at the same time matching *in vivo* flow conditions.

Also for the LV, three characteristic peaks were observed, representing ventricular emptying during systole and the early and late filling phase during diastole. Again, the CT-based simulations showed a good agreement with the 4D Flow MRI measurements. Contrary to the LA, the DoE simulations showed negligible KE variations—clearly the effect of inlet PV flow rate appeared to be low on LV flow energetics. The LA and LV KE levels agreed well with *in vivo* measurements and to average values found in other studies.^2^,^3^,^11^,^18^

### Mitral Valve Flow Profiles

**Figure 4:**
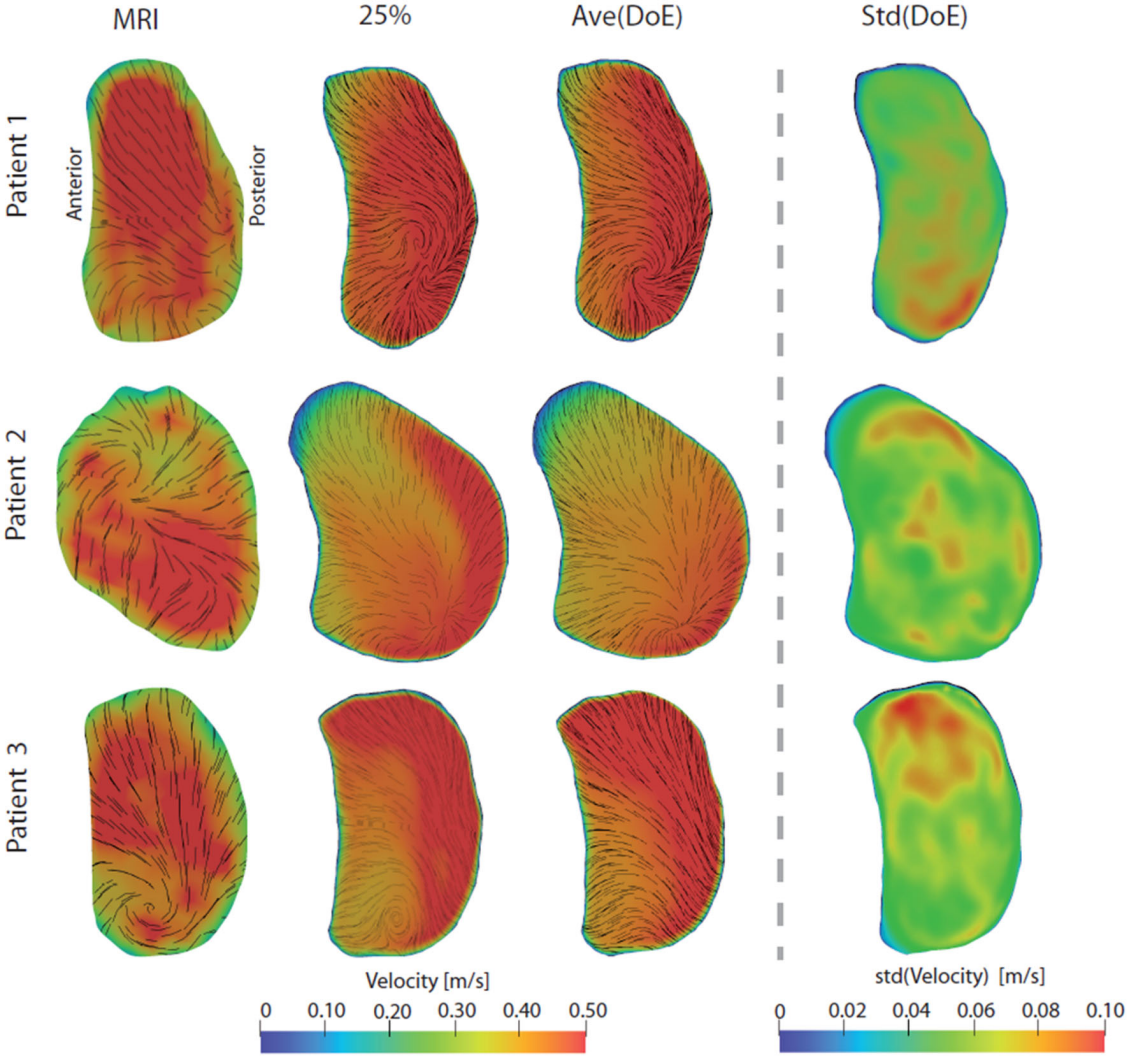
Mitral valve flow profiles during early filling. From left to right: *in vivo* 4D Flow MRI measurements, CT-based simulation with 25% of the total flow entering each PV in the LA, average flow profile for all 20 DoE simulations, and standard deviation of all 20 DoE simulations. The velocity magnitude is indicated by color, while short streamlines show in-plane vortical motion.

**Figure 5:**
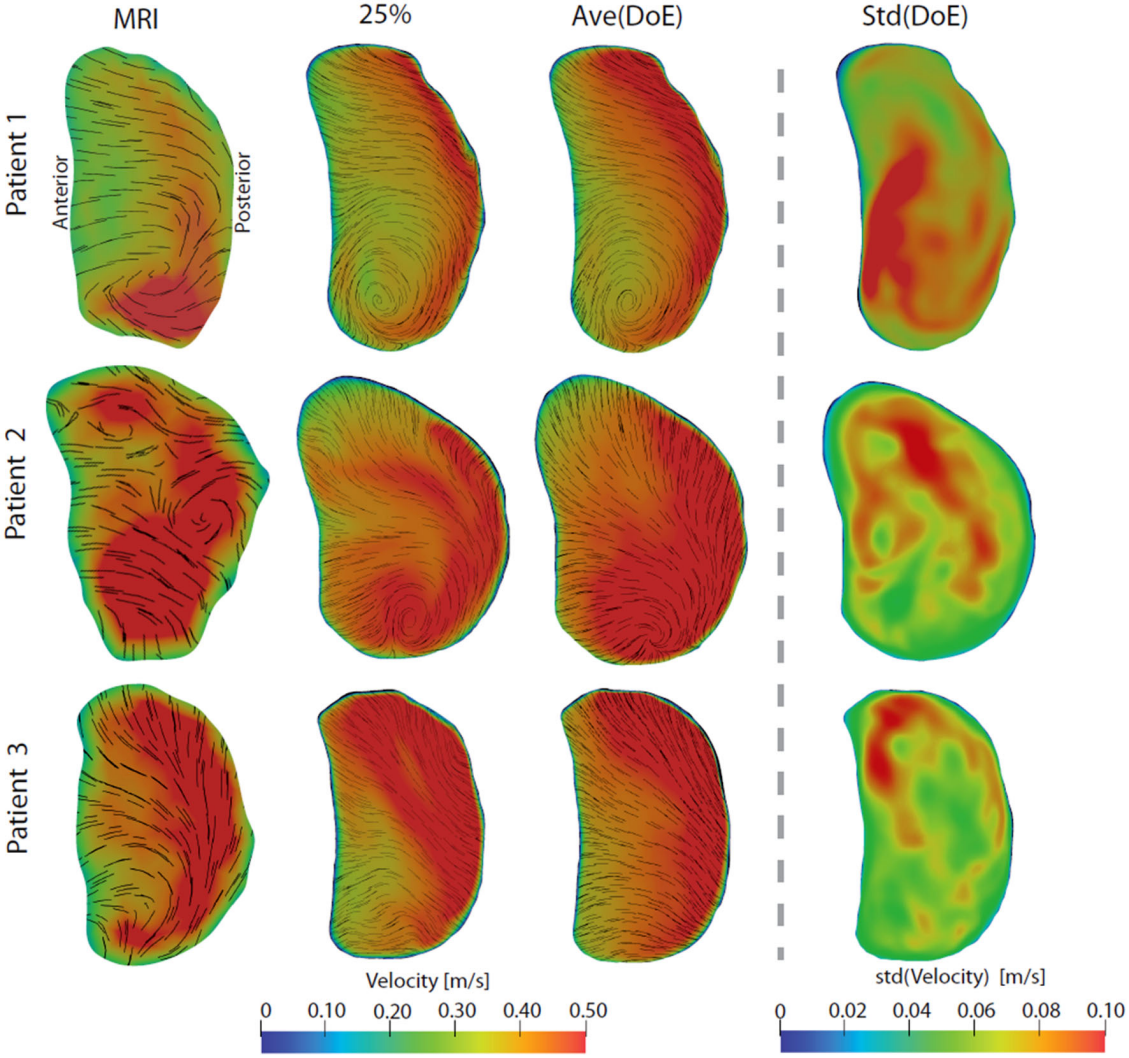
Mitral valve flow profiles during late filling. From left to right: *in vivo* 4D Flow MRI measurements, CT-based simulation with 25% of the total flow entering each PV in the LA, average flow profile for all 20 DoE simulations, and standard deviation of all 20 DoE simulations. The velocity magnitude is indicated by color, while short streamlines show in-plane vortical motion.

To assess any regularizing effect of the mitral valve, flow profiles at early and late diastolic filling were assessed, see Figs. 4 and 5. The early filling flow profiles were generally blunt with some local in-plane swirling motion, revealing vortical structures still present in the mitral jet. Qualitatively, the CT-based flow profiles were similar to the *in vivo* measurements, with both similar velocity magnitude and in-plane motion. The average flow profile of all DoE simulations was similar to the 25% simulation, and a standard deviation <0.10 m/s for all 20 DoE simulations in each of the three patients indicated that the effect on pulmonary vein flow rates were low on the mitral jet profile. Generally, the late filling mitral valve flow profiles are a bit more skewed, with more flow towards the posterior side. Vortical in-plane motion were again observed for both MRI measurements and simulations, and compared to the early filling flow profile, larger variations were observed among the 20 DoE simulations indicated by the standard deviation profile.

### Root Mean Square Error Analysis

**Figure 6:**
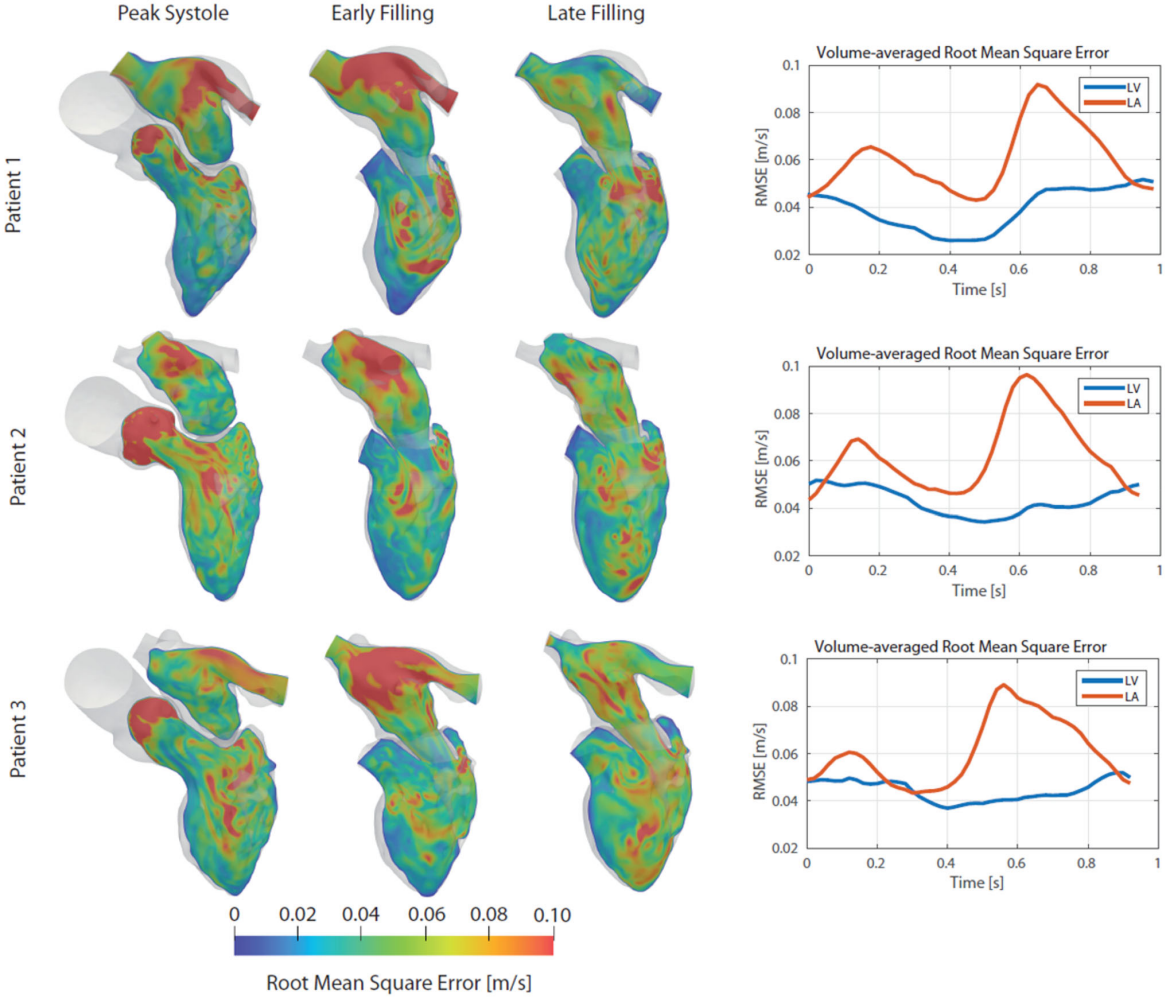
Cross-sectional plane covering the LA and LV showing root mean square errors of velocity for the 20 DoE simulations at peak systole, early and late diastolic filling. Additionally, the RMSE was volume-averaged in the LA and LV over the entire cardiac cycle, as shown in the panels to the right.

To assess the effect of pulmonary vein inflow on atrial and ventricular flow patterns, root mean square errors were calculated using DoE #1 (with *f *= 25%) as baseline. Contour plots of RMSE are presented at peak systole, early filling and late filling, together with quantified volume-averaged RMSE for the LA and LV over the entire cardiac cycle in Fig. 6. At peak systole, elevated values of RMSE were found in the ascending aorta, due to the acceleration and expansion of the flow after the aortic valve in the aortic sinuses. Peak systole and early filling coincide with the bi-phasic filling of the LA, and large RMSE were present in the LA, as variations in inflow rates will result in notably different LA flow fields. The mitral jet profile had low RMSE values for all three patients during the early filling phase, consistent with the low standard deviation values for the mitral flow profile in Fig. 4. When the flow was pulled through the mitral valve it became regularized, with only minor variations.

However, the flow in characteristic mitral valve vortex was affected, as elevated RMSE vales were present in the direct vicinity of the valve leaflets in the LV. Velocity magnitude contour plots for all 20 DoE simulations for the three patients are presented in the appendix. The largest RMSE values were observed in the LA during the bi-phasic filling, at peak systole and early diastolic filling. The flow from each PV will collide, and depending on flow rate, will create different LA flow patterns. The LV had lowest RMSE values at the end of systole as the remaining LV flow is mostly quiescent, before the LV starts to fill again.

## Discussion

In this study, the effect of variation in pulmonary vein inflow rates on cardiac flow patterns was investigated in a systematic manner using numerical simulations in a design-of-experiment approach. Three patients with suspected heart disease were studied, and results were compared to patient-specific *in vivo* 4D Flow MRI measurements. From a modeling perspective, a sensible approach in the absence of *a priori* knowledge about the flow distribution in the PVs would be prescribing an equal flow through each PV. Results showed that while different PV inflow rates affect the LA flow patterns, a regularizing effect by the mitral valve removes much of the flow asymmetry created in the LA when the blood enters the LV.

Intra-atrial KE levels were found to be strongly affected by PV inflow rates. Large variations were found, especially during the early filling phase in diastole when blood is drawn into the LV from the LA. Generally, the approach with equal amount of flow entering the LA through all four PVs resulted in the lowest atrial KE values, possible due to similar inflow velocities; for any other inflow combination the local velocity would increase on at least one PV and as the KE scales as the square of velocity, the intra-atrial KE would also increase. Even though the flow distribution was not divided equally among the four PVs in the *in vivo* MRI data, and measured flow rates and flow volumes did not perfectly match the CT-based simulations, both KE levels in the LA and LV, as well as mitral valve profiles agreed well between the two modalities. *In vivo* MRI measurements showed consistently higher flow volumes for the right side PVs than the left side, which is sensible as there are normally three lobes in the right lung, but only two in the left. A number of DoE cases represents extreme cases where most of the flow enters through only two PVs (e.g., case #6, #9 and #16). While these affect atrial flow patterns and energetics, the effect on mitral valve profiles and ventricular kinetic energy was still minimal. In-plane vortical motion was observed in the mitral flow, suggesting that some of the vortical structures created in the LA could be transferred to the LV. These structures mainly affected the vortex created in the direct vicinity of the mitral valve, while the remaining flow field in the LV was less affected. Based on the RMSE computations on the DoE analysis, the average difference in flow velocity is higher in the LA than in the LV, further highlighting the regularizing effect by the mitral valve.

Abnormal flow patterns in the LA could potentially initiate thrombus formation in the LA and LAA,^1^,^16^,^17^,^21^ predominantly in regions of high blood residence time. Furthermore, it has been suggested that asymmetric filling of the LA would preserve momentum as rotating flow structures are redirected towards the atrio-ventricular valves.^19^ However, the importance of momentum preservation is debated,^29^ as the KE levels of the flow are orders of magnitude lower than the external work done by the LV on the blood during systole at rest.^12^ In this study, we found that even though the KE levels were different in the LA due to different PV inflow rates, the LV KE levels were unaffected. This is arguably due to a regularizing effect of the mitral valve. Furthermore, simulation results also showed that even though PV inlet flows were different, mitral valve velocity profiles were similar with low variation for all cases. This was expected as the flow was accelerated through the mitral orifice as it is drawn into the LV, and normally acceleration tend to have stabilizing and regularizing effect on the flow. While elevated RMSE values were observed in the LA due to the different flow patterns, low RMSE values were observed in the mitral jet, due to the flow acceleration. However, small perturbations and vortices could still remain which was also observed in the current results; directly after the mitral leaflets when the flow was allowed to expand in the LV, the characteristic mitral valve vortex in the LV was affected by small variations still present in the mitral jet, as indicated by the elevated RMSE values. However, large scale flow features were still unaffected.

The computational models in this study included both papillary muscles and LV trabeculae, as it has been shown to affect intraventricular flow patterns.^23^ Models used in earlier studies are often significantly smoothed due to either insufficient image resolution or the high computational cost associated with the geometrical complexity in the model. In this study, the spatial resolution of the CT acquisition (0.3 mm) was an order of magnitude higher than the 4D flow MRI (2.9 mm), which are typical resolution for these cardiac acquisitions.^10^ The flow volumes were consistently higher for the *in vivo* flow measurements than the CT-based simulations. While the difference in spatial resolution may explain some of these findings, we expect that the main reason for this difference is that the CT and MRI data were acquired with different breathing techniques. The MRI acquisition was the average of several hundreds of heart beats during free breathing, and acquired with a breathing navigator at an end-respiratory phase, whereas the CT acquisition was performed during an inspiration breath hold over 7–10 s. Venous return to the right side increases during inspiration, which will decrease left ventricular filling and stroke volume by means of interventricular interaction. Similarly, LV stroke volume is normally higher during expiration, and together these two mechanisms could explain differences in stroke flow volumes. As the flow volumes were different for the MRI measurements and CT-based simulations, no attempts to prescribe *in vivo* measurements in the simulations were performed. Rather, the differences in flow volumes extracted from the two imaging modalities highlight the difficulties associated with cardiac modeling.

Using CT-data, the wall motion was prescribed in the simulation model. This meant that momentum was passed from the wall to the blood, but the blood flow dynamics were unable to affect the prescribed wall motion. This one-way transfer of momentum could potentially have an adverse effect on the computed flow patterns, as hemodynamic forces could affect cardiac geometry and motion locally. Similarly, the dynamics of the mitral valve leaflets was not prescribed due to limitations in temporal resolution of the CT acquisition. However, the leaflets were allowed to move with the valve plane and the mitral orifice diameter changed over the cardiac cycle. Improved mitral valve leaflet dynamics may affect the LV flow patterns and the strength of the regularizing effect seen in this study. As the wall motion is prescribed from measurements, the simulation method requires a retrospective CT acquisition covering the entire cardiac cycle.

Blood flow residence time was not assessed, due to the high computational cost associated with particle tracing. To obtain reliable statistics on residence time, a large number of particles would need to be tracked over several cardiac cycles. In this study we performed 20 different simulations for each of the three patients, and the computational cost of assessing residence time was considered to be too high.

In conclusion, by using a large number of different numerical simulations in a systematic manner it was found that while atrial flow patterns were significantly affected by pulmonary vein flow rates, the mitral valve regularizes the flow and only minor effects could be observed in the ventricle. Comparing simulated flow fields to *in vivo* flow measurements showed that equal flow through each PV agreed well.

## Electronic supplementary material

Below is the link to the electronic supplementary material.

Supplementary material 1 (PDF 5585 kb)
