# Supplementary material for: Impact of Pulmonary Venous Inflow on Cardiac Flow Simulations: Comparison with In Vivo 4D Flow MRI
Source: Ann Biomed Eng. 2018 Oct 24;47(2):413–24. doi: 10.1007/s10439-018-02153-5 (PMC6342898; doi:10.1007/s10439-018-02153-5)
Supplement: Supplementary file 1 — Supplementary material 1 (PDF 5585 kb) [file 10439_2018_2153_MOESM1_ESM.pdf]

Appendix

Velocity magnitude contour plots for all 20 DoE simulations for the three patients.

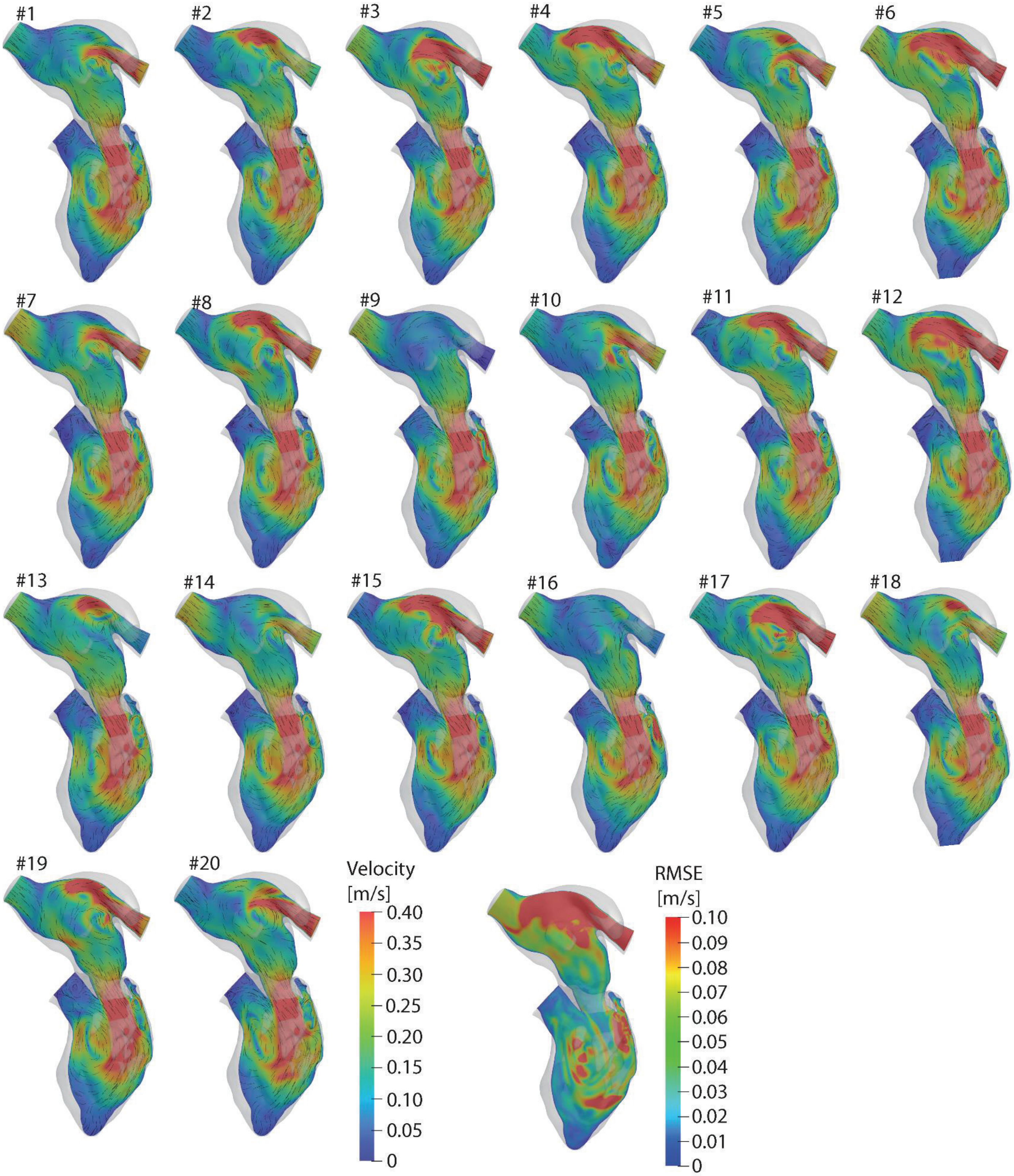

Velocity magnitude contour plots for all 20 DoE simulations for patient 1.

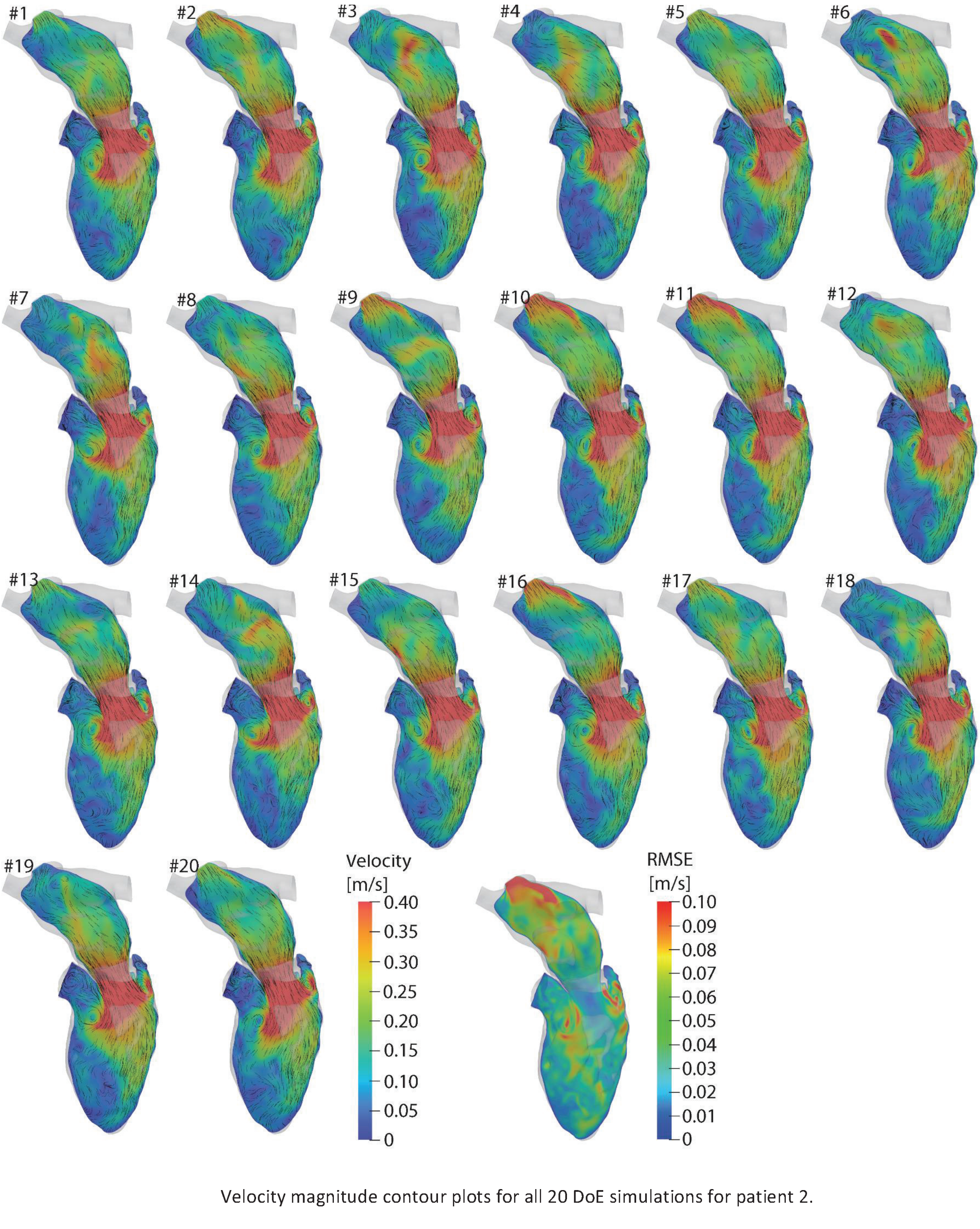

Velocity magnitude contour plots for all 20 DoE simulations for patient 2.

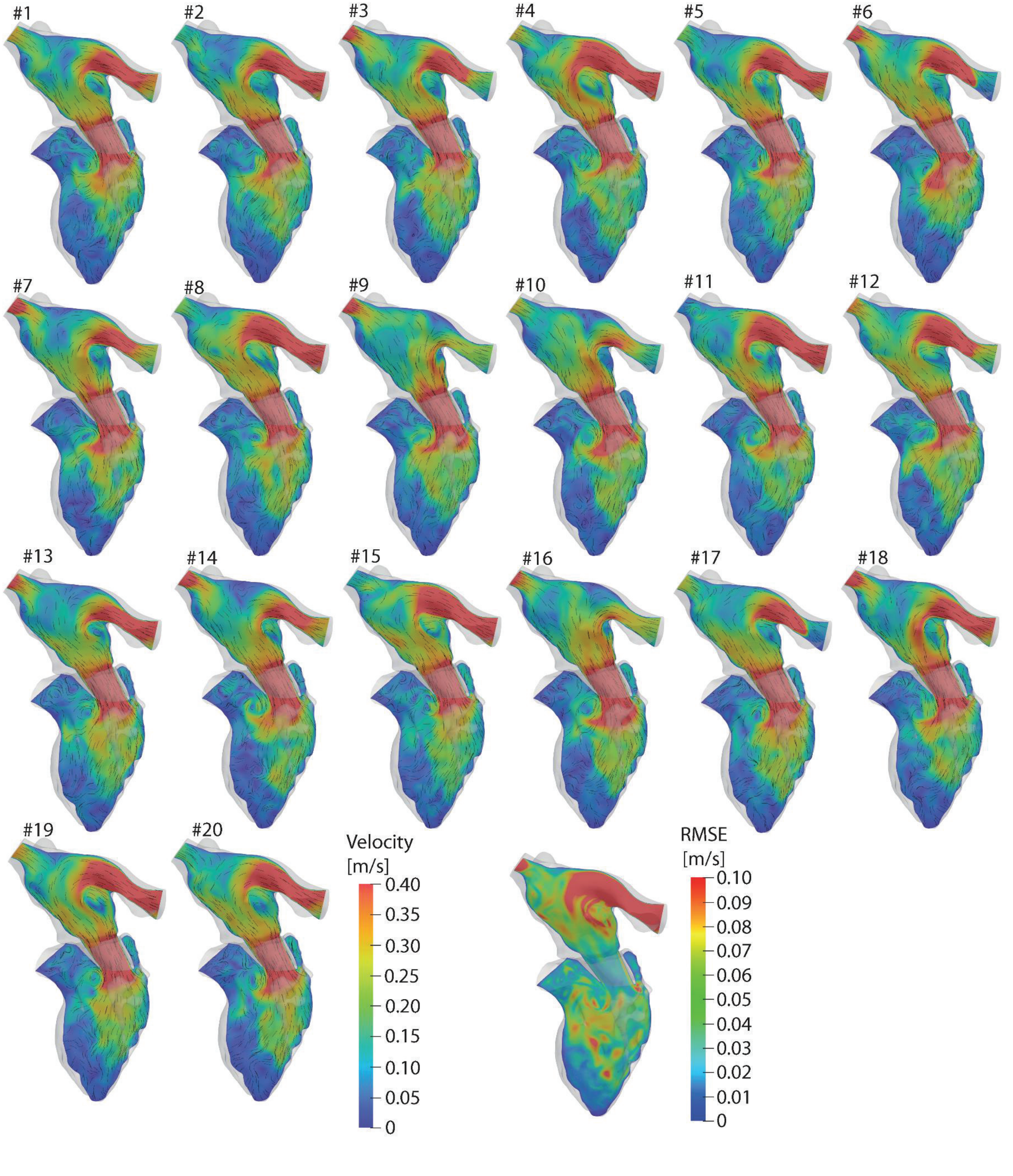

Velocity magnitude contour plots for all 20 DoE simulations for patient 3.
